# Sexual Dimorphism in Plantar Pressure Distribution Patterns

**DOI:** 10.33549/physiolres.935648

**Published:** 2025-12-01

**Authors:** Jacek LORKOWSKI, Adam JÓŹWIK, Mieczyslaw POKORSKI

**Affiliations:** 1 Center for Vision, Speech and Signal Processing, University of Surrey, Guildford, UK; 2 Department of Management and Accounting, SGH Warsaw School of Economics, Warsaw, Poland; 3 Faculty of Physics and Applied Computer Sciences, Lodz University, Lodz, Poland; 4 Institute of Health Sciences, Opole University, Opole, Poland

**Keywords:** Biomechanics, Foot, *K*-nearest neighbors’ algorithm, Pedobarography, Plantar pressure, Foot dimorphism

## Abstract

Efficient management of foot disorders requires knowledge of plausible sexual dimorphic differences in the foot structure and function. Knowledge about foot dimorphism is yet unsettled. In the present study, we mapped the multizone plantar pressure distribution in a cohort of 298 healthy subjects of both sexes using the piezoelectric pedobarographic recordings. We investigated the hypothesis that sexual foot dimorphism would entail differences in plantar pressure distribution. We delved into the issue using the k-nearest neighbors’ classifier (k-NN), a supervised machine learning algorithm, to predict the correct foot phenotype classification based on the plantar pressure and anthropometric and structural features. Based on the similarity measures, the classifier assigns unknown input data to the most common class, men or women, among the closest neighbors. The major finding was the unraveling of sex-specific foot dimorphism in healthy people. The k-NN was able to recognize the person’s sex based on a few discriminatory features, with an outstandingly low misclassification rate. The pressure distribution on the plantar surface of the great toe, one of the orthopedic plantar surface zones, is the most distinct feature in differentiating the foot phenotype. The results also unraveled the presence of individuals whose foot features paradoxically corresponded with those classified for the opposite sex, i.e., the female foot was classified in the men’s group and vice versa. In conclusion, the study supports the presence of sexual foot dimorphism. A pedobarographic examination supplemented with the k-NN algorithm for machine learning allows one to discriminate the foot sexual phenotype with high accuracy. The foot phenotype identities exist outside the gender binary. Insights into sexual foot dimorphism may streamline the management of foot disorders.

## Introduction

Sex differences in bodily systems are well recognized [1,2]. However, knowledge about differences in structural foot buildup is scarce and limited to the kinematics of the foot. There are reports that the strain of plantar fascia and the stiffness of the heel pad are greater in females than in males, which predisposes the former to fasciitis and foot trauma, particularly in loaded conditions of sporting injuries [3–5]. These reports do not translate into knowledge about differences in plantar pressure distribution.

Assessing underfoot zonal pressure distribution is germane for the diagnosis of foot disorders. Several plantar surface divisions have been created in recent decades based on anatomical and orthopedic foot segments [6]. These divisions differ in the number and position of specific zones over the plantar surface. The rationale for choosing a division in patient management lacks a verifiable, evidence-based background. However, there is a consistent impression that a multizone plantar division has an edge over a simple segmental one because it holds more detailed information on the underfoot pressure distribution. Translation of the plantar pressure departure from the pattern present in healthy feet into the efficient management of foot disorders requires knowledge of plausible differences in the pressure distribution between men and women and between the right and left foot. These essential control pieces of information have not yet been tackled systematically.

Here, we mapped the plantar pressure network in a cohort of healthy subjects. We assessed static foot-ground pressure points using Lorkowski’s plantar surface division into 12 zones in six segmental parts [7]. We tested the hypothesis that healthy foot plantar pressure distribution would inherently differ between the sex phenotypes. The aim was to develop an algorithm that recognizes the foot phenotype based on features selected from anthropometric characteristics, foot-sidedness, and plantar pressure distribution. To this end, we used the k-nearest neighbors (k-NN) classifier, a supervised machine learning algorithm, a subset of artificial intelligence, to predict the correct phenotype classification.

## Methods

### Participants and plantar pressure examination

This was an observational cohort study. It was performed in accordance with the principles of the Declaration of Helsinki for Human Research, its later amendments, and the standards of the national legislation for human research. The Bioethics Committee at the District Medical Chamber in Krakow, Poland (no. OIL/KBL/18/2019) granted permission to conduct the study. Informed consent was obtained from each study participant. Participants enjoyed full anonymity.

Two hundred ninety-eight Caucasians, aged 18–60 years, scheduled to undergo routine ambulatory medical check-ups (male/female - 141/157), were recruited for pedobarographic examinations. Male and female participants did not differ significantly in age and were used to performing low-to-moderate physical activity daily. They had no history of acute or chronic ailments, normal results of physical examinations, including the function of the locomotor system, and no ankle or foot complaints or disorders during the year before the examination. Subjects underwent examinations on a PEL 38 multipoint 1024-piezoelectric sensor plantar-force transducer platform and Twin 99 software (Medicapteurs, Toulouse, France). The platform dimension was 515×445 mm with an active area of 320×320 mm. The examination was performed in an upright externally unsupported standing position for 30 seconds, with arms down, without movement, and with bare feet closely sticking to the platform’s surface. The test was repeated three times at 3-minute intervals, and an average value was taken for further processing. The platform had 500 pressure pick-up points, which enabled the elucidation of regularly distributed zonal pressure points in both foot support areas. The pressure was expressed in g/cm^2^ and then processed into the percentage values of pedobarographic parameters.

### Statistical elaboration

General demographic and plantar continuous data were expressed as means ±SD. The inter-sex general and foot-side corresponding data, and between the two feet in each sex, were compared with the two-tailed Mann-Whitney U test. A p-value of less than 0.05 marked significant differences, meaning a 5 % chance of incorrectly rejecting the null hypothesis of no changes between men and women.

### K-Nearest neighbors’ (k-NN) algorithm

**Fig. 1:**
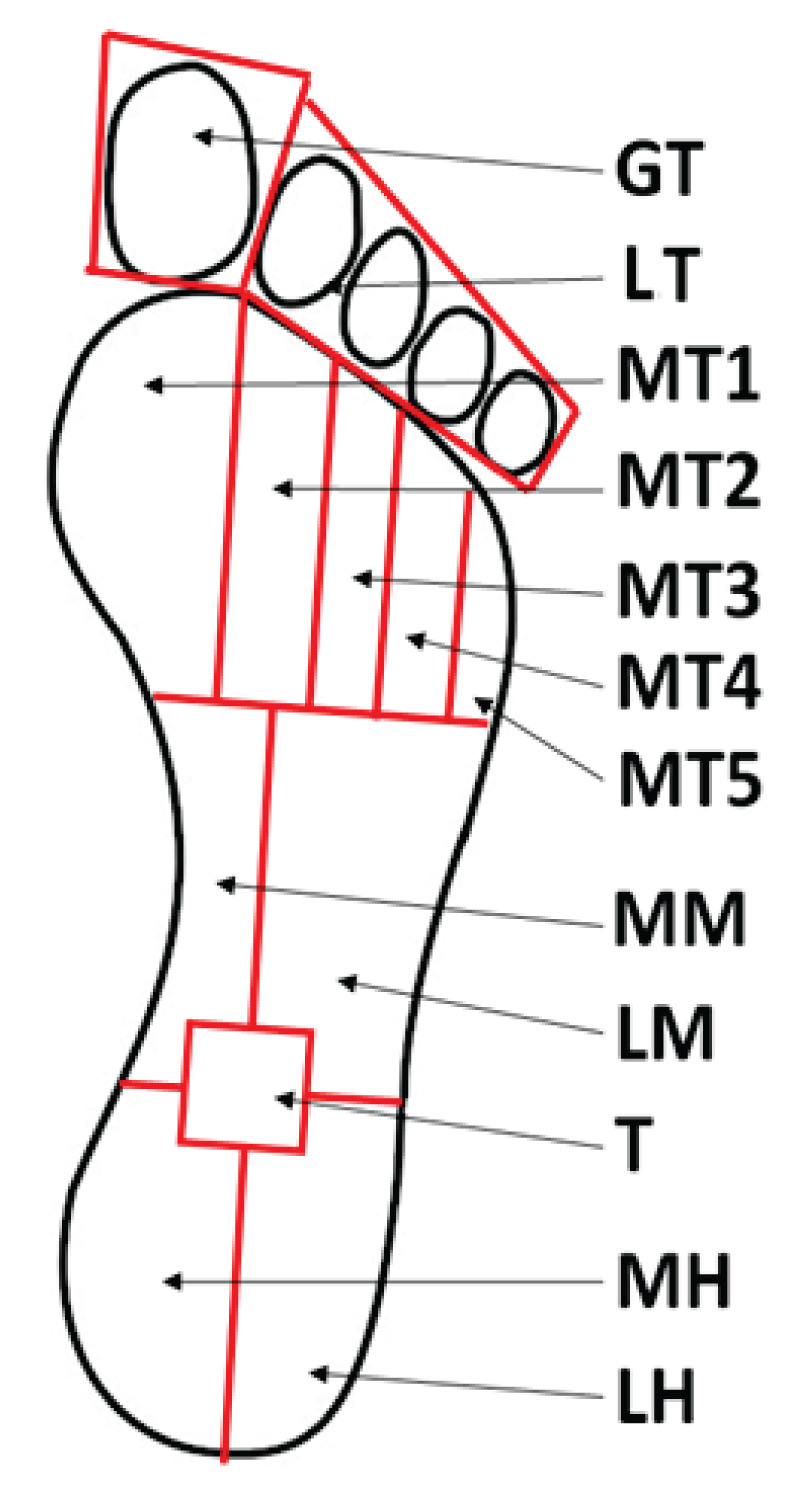
Anatomical division of plantar surface into zones: GT - great toe, LT - lateral toes, MT1 to MT5 - metatarsal 1st to 5th, MM - medial midfoot, LM - lateral midfoot, TA - tarsal, and MH - medial hindfoot, LH - lateral hindfoot; according to Lorkowski [7].

The analysis was based on a division into Class 1 - men and Class 2 - women. Conglomerates of seventy features and plantar pressure points were assessed in each subject of either class, including the subjects’ demographics and the left and right plantar pressure points. The conglomerates included sets of five pressure points corresponding to each of the twelve plantar zones depicted in Fig. 1. These points were alike in each zone and expressed in absolute and percentage-processed terms. The great toe (GT) zone below exemplifies the content of the pressure points:

- Average pressure under the great toe in absolute terms (GT-AvP, g/cm^2^).
- Average pressure under the great toe as a percentage of the maximum pressure recorded in the two feet of each participant - GT-AvP (%MAX),
- Maximum pressure under the great toe in absolute terms (GT-MxP, g/cm^2^).
- Maximum pressure under the great toe as a percentage of the maximum pressure recorded on the two feet of each participant- GT-MxP (%MAX).
- Minimum pressure under the great toe as a percentage of the maximum pressure recorded in the two feet of each participant - GT-MiP (%MAX).

A complete list of average plantar pressure data recorded in the orthopedic zones was provided in an Excel Sheet as Supplementary material.

Given the datasets with the two different classes, we used the k-NN algorithm with the leave-one-out cross-validation, which offers the highest correct classification rate of test data [8–10]. The algorithm requires a reference set of objects, called the training set, with the known class membership, to predict the correct class to which the test data belong by calculating the distance between the test data and all the training points. The test data totaled 70 demographic and plantar pressure features and attributes.

The features of the reference dataset can be strongly correlated or redundant, which may increase the error rate. Therefore, the feature selection is recommended. To assign the foot to the person’s sex, the algorithm selects the *k* points closest to the test data and assigns them to the class most heavily represented among its k-nearest neighbors in the reference set, where *k* is a parameter set up experimentally. We searched for the *k* offering the lowest misclassification rate using the leave-one-out validation, in which the reference set was reduced each time by one person, the one whose sex was being recognized. The k-NN classifier was supplemented by a 2×2 confusion matrix presented in Table 1. This table corresponds to a contingency table, which enables calculating the significance level for the association between two qualitative variables: the true class and the assigned class. The class assigned stands for the “voice of features”. Thus, the significance level for the dependence between the true and assigned classes can be taken as the one between the features and the classes [11].

## Results

### Plantar pressure assessment

Table 2 shows an overview of demographics and sex-split plantar pressure features. The age was closely comparable in both sexes. All other anthropometric and plantar pressure features were significantly lower in women than in men, although the differences were of a small 4–10 % range. Similar differences were seen in women when comparing the left or right foot with those of men. The average maximum pressure (MAX) recorded in the two feet of each participant was a major reference point for further statistical elaboration; it was 5.5 % lower in women (p<0.05). The average pressure (AvP) and the maximum pressure (MxP) for the left and right foot, presented in arbitrary units, were significantly lower in women than in men. These sex differences became insignificant when the former was expressed as a percentage of MxP and the latter as a percentage of MAX. An across-the-board tendency for slightly lower plantar pressure values in women than men was noticeable in each of the twelve plantar zones from the great toe down to the heel area, irrespective of the foot side. This tendency notably concerned the metatarsals (see Supplementary Material for detailed figures). Given the meager and variably zone-scattered changes, the inter-sex comparisons of the sheer levels of the plantar pressure were baseless. Instead, we used the k-NN algorithm to predict the foot-sex phenotype based on the key feature classification.

### K-Nearest Neighbors (k-NN) Assessment

Tables 3 and 4 show k-NN results for the left and right foot, respectively. The selection of features was contingent on the forward feature review. The best number of *k* was determined for each feature combination.

For the left foot, the k-NN algorithm assigned 13 out of the 141 men to the women’s group and vice versa, three out of the 157 women to the men’s group, based on the two selected features: FL and GT-AvP (%MAX), with a prediction misclassification rate of 5.4 %. There was a 9.2 % probability that a man was assigned to women and a 1.9 % probability that a woman was assigned to men.

For the right foot, the algorithm assigned 13 out of the 128 men to the women’s group and vice versa, one out of the 157 women to the men’s group, based on the five selected features: FL, FA, GT-AvP, and MH-AvP (%MAX), with a prediction misclassification rate of 4.7 %. There was a 2.3 % probability that a man was assigned to women and a 7.8 % probability that a woman was assigned to men.

## Discussion

This study was designed on the empirical premise that the plantar pressure distribution in healthy feet may differ between men and women, which may bear on foot morbidity. To resolve the issue, we performed pedobarographic investigations of the plantar pressure in foot-healthy adults of both sexes. The investigation was based on pressure measures using a plantar-force transducer platform with in-built piezoelectric sensors. The pressure distribution points were mapped out in the twelve zones of the plantar surface according to Lorkowski’s zone division [7]. Here, we delved into sexual dimorphism using the k-NN algorithm, a supervised learning classifier, to predict the correct foot phenotype. The major finding was that the inter-sex plantar pressure distribution differed. We found two highly sex-discriminatory features for the left foot and five for the right foot, with a low misclassification rate of sex recognition of 5.4 % and 4.7 %, respectively. Intriguingly, the results unraveled the presence of several individuals paradoxically assigned to either classifier’s configuration, i.e., the female foot was classified in the men’s group and vice versa, meaning that the foot features corresponded with those of the opposite sex. Thus, the foot phenotype framework should include the features of both men and women rather than have a binary character.

Moreover, the findings point out that the pressure in the segmental orthopedic zones of the plantar surface is lower in women than in men. The differences were of a small 5–10 % range, but statistically significant. Lower plantar pressures in women than in men were seen in the side-corresponding feet, with no appreciable differences between the two sides in each sex.

The underlying reasons for sexual foot dimorphism could be related to the evolutionarily shaped female body structure, limb musculature and motion, foot size, and, on average, about 15 % lower body mass compared to men [12]. Studies show that US adult women, on average, are 9 % shorter and 16.5 % lighter than men [13]. Women are about 40 % less strong than men in their upper bodies and 80–90 % less strong in their lower bodies. These differences center around higher testosterone levels in men, which correlate with muscle mass and strength, and larger average anthropometric parameters [14]. Such sexual dimorphism of body stature aligns with the anthropometric parameters reported in the present study. However, knowledge of where a healthy foot is situated in the overall dimorphic evolutionary picture of physical differences is scarce. The available data mostly pertain to the footprint dimensions shaping the foot silhouette that might factor in the sex determination. Foot dimensions are ethnicity-related and closely depend on the individual’s body structure, and as such, they have poor sex acumen [15–17]. The underlying mechanisms of the downward plantar pressure changes in women could not be settled in the present study, as the issue would require alternative study designs.

The use of foot parameters for sex prediction has scarcely been investigated. The present study found that some foot features predominated in the k-NN sex prediction using either foot. These features were the foot length and the pressure contact force exerted by the great toe. This somehow refers to the earlier studies of Suleiman *et al.* [16] who showed that the left foot breadth and right foot length were the best sex determinators, and Krishan *et al.* [17] who showed that the foot length ratios of the great toe to successive small toes, i.e., T1/T2, T1/T3, etc., showed distinct sex differences. Those studies, which pertain to forensic procedures, have ethnic limitations since they were performed in Nigerian and Indian populations, respectively. Nonetheless, the great toe and its anatomic joint system are prominent players in common foot disorders and deformities such as arthritis, hallux vulgaris, or hallux rigidus, and sports and motion activities that rely on the body’s musculoskeletal axis [18,19]. Moreover, the contact pressure of the great toe plays a role in the pathophysiology of gait since the compressive pressure on the knee increases when the knee is transferring forward over the toe more while bending in walking. The present study broadens the importance of the great toe by adding its role in finding foot dimorphism.

This study has several limitations. The brain’s proprioceptive and coordinative aspects of sexual dimorphism of the musculoskeletal axis could not be elucidated. It is still unclear whether the foot sex-opposite features in either class are a lone biological incidence of local foot shape and osteology, or they are accompanied by more sexual dimorphism, i.e., connected with the inheritance of other sexual patterns, which could affect the shaping of an individual’s life experiences and opportunities. The resolution of these issues would require alternative study designs. Further, the k-NN classifier has the disadvantage of assuming equal importance to all features, which makes it sensitive to irrelevant parameters. Additionally, the parameters we chose, despite being pedobarographic indices, had a degree of subjectivity. However, on the k-NN’s beneficial side lies its simplicity, lack of assumptions about data, ability to apprehend complex interactions among variables, and high ability to make good predictions.

## Conclusions

This study lends support to the existence of foot sexual dimorphism in healthy people. Combining a pedobarographic examination with the k-NN algorithm for the machine learning method allows for the discrimination of the foot sexual phenotype with high accuracy. Intriguingly, the pressure distribution on the plantar surface of the great toe, one of the orthopedic plantar surface zones, is an outstanding distinguishing feature of the phenotype setting in both left and right feet. The study shows that the k-NN algorithm differentiates individuals whose foot features correspond with those of the other sex. Thus, a tendency to reduce the foot phenotype to the sex-specific binary framework may lead to ambiguous consequences. This study has deepened insights into sexual foot dimorphism, which may help manage foot disorders.

## Supplementary Information



## Figures and Tables

**Table 1:** Confusion matrix for two-class classification.

	Assigned Class 1	Assigned Class 2
*True Class 1*	n [1, 1]	n [1, 2]
*True Class 2*	n [2, 1]	n [2, 2]

*n*, number of subjects

**Table 2:** Demographic and general plantar pressure parameters

	Men (n=141)		Women (n=157)	
*Age (yr)*	50.5 ±1.2		48.8 ±1.1	
*BM (kg)*	72.5 ±7.1		61.0 ±6.2†	
*BH (cm)*	176.6 ±5.8		165.3 ±5.6†	
*BMI (kg/m* *^2^* *)*	23.2 ±1.5		22.3 ±1.7†	
*MAX (g/cm* *^2^* *)*	560.3 ±106.4		529.3 ±96.2†	
*FL (cm)*	27.0 ±1.0		24.5 ±0.8†	

	**Left foot**	**Right foot**	**Left foot**	**Right foot**

*FA (cm* *^2^* *)*	132.4 ±14.5	134.9 ±16.0	116.5 ±5.0*	117.3 ±12.3**
*FT (%)*	50.2 ±3.0	49.8 ±5.1	50.8 ±2.8	49.2 ±2.8
*AvP (g/cm* *^2^* *)*	284.4 ±47.9	276.1±47.4	264.5±43.5*	253.8 ±40.1**‡
*AvP (% MAX)*	51.1 ±4.5	49.7 ±6.1‡	50.4 ±5.3	48.4 ±4.8‡
*MxP (g/cm* *^2^* *)*	547.7 ±104.5	528.4 ±97.6	515.7 ±98.0*	498.8 ±92.2**
*MxP (% MAX)*	97.8 ±4.8	94.6 ±6.7‡	97.4 ±4.9	94.4 ±6.6‡

Data are means ±SD. BM, body mass; BH, body height; BMI, body mass index; MAX, average maximum plantar pressure recorded in a sex group, irrespective of foot-sidedness; FL, foot length; FA, foot surface area; FT, foot thrust - foot’s surface area as a percentage of both feet’s area; AvP, average underfoot pressure; MxP, underfoot maximum pressure;

† p<0.05 for the general inter-sex differences,

* p<0.05 for the corresponding left foot in the opposite sex,

** p<0.05 for the corresponding right foot in the opposite sex, and

‡ p<0.05 between the two feet in the same sex.

**Table 3:** Left foot dimorphic phenotype - k-NN classification.

	Assigned to Men	Assigned to Women	
*Men (n)*	128	13	p<0.0001
*Women (n)*	3	154	

Optimum *k*-NN = 4. Selected features: FL and GT-AvP (% MAX)			
Misclassification error rate = 0.054			

	**Probability to be assigned to Men**	**Probability to be assigned to Women**	

*Men (n)*	90.8 %	9.2 %	
*Women (n)*	1.9 %	98.1 %	

*n*, number of subjects; *p* denotes the significance between classes and features connectivity; FL, foot length (cm); GT-AvP (%MAX), average pressure under the great toe as a percentage of the maximum pressure recorded in the two feet of each participant.

**Table 4:** Right foot dimorphic phenotype - *k*-NN classification.

	Assigned to Men	Assigned to Women	
*Men (n)*	128	13	p<0.0001
*Women (n)*	1	156	

Optimum *k*-NN=10. Selected features: FL, FA, GT-AvP, GT-AvP (%MAX), and MH-AvP (%MAX)			
Misclassification error rate = 0.047			

	**Probability to be assigned to Men**	**Probability to be assigned to Women**	

*Men (n)*	97.7 %	2.3 %	
*Women (n)*	7.8 %	92.2 %	

*n*, number of subjects; *p* denotes the significance between classes and features connectivity; FL, foot length (cm); FA, foot surface area (cm^2^); GT, great toe; AvP, average underfoot pressure (g/cm^2^); AvP (%MAX), average pressure as a percentage of the maximum pressure recorded in the two feet of each participant; MH-AvP (%MAX), average under-heel medial pressure as a percentage of MAX.
