# Supplementary material for: Sexual Dimorphism in Plantar Pressure Distribution Patterns
Source: Physiol Res. 2025 Dec 1;74(6):999–1005. doi: 10.33549/physiolres.935648 (PMC12721814; doi:10.33549/physiolres.935648)
Supplement: Supplementary file 1 [file Lorkowski-SupplementaryMaterial.pdf]

Sexual Dimorphism in Plantar Pressure Distribution Patterns

Jacek LORKOWSKI[1, 2], Adam JÓŹWIK[3], Mieczyslaw POKORSKI[4]

Supplementary Material

A list of plantar pressure average data recorded in the orthopedic zones of the left and right foot in men and women alike.

	Class 1 - MEN (141)											
	MAX (g/cm^2) - 560.3 ±106.4											
	Left Foot						Right Foot					
	Plantar pressure data						Plantar pressure data					
Plantar zone	MxP (g/cm^2)	MxP (%MAX)	AvP (g/cm^2)	AvP (%MAX)	MnP (g/cm^2)	MnP (%MAX)	MxP (g/cm^2)	MxP (%MAX)	AvP (g/cm^2)	AvP (%MAX)	MnP (g/cm^2)	MnP (%MAX)
GT	224.3 ±73.5	41.2 ±14.7	156.1 ±48.5	28.4 ±8.9	86.8 ±31.2	15.6 ±4.4	221.5 ±78.6	41.3 ±17.1	151.4 ±47.3	27.9 ±9.6	84.7 ±25.3	15.2 ±3.7
LT	156.2 ± 75.9	29.1 ±14.7	116.6 ±54.5	21.6 ±10.1	79.7 ±44.4	14.7 ±7.5	143.1 ±83.4	26.9 ±16.9	107.5 ±58.5	20.0 ±11.3	73.8 ±42.3	13.5 ±7.4
MT1	370.5 ±71.4	67.2 ±12.2	264.4 ±50.6	47.9 ±8.3	101.1 ±38.5	18.2 ±5.7	355.5 ±61.9	64.8 ±12.3	246.2 ±40.6	44.8 ±7.7	99.8 ±32.2	17.9 ±5.1
MT2	397.7 ±73.0	71.9 ±11.4	310.5 ±55.9	56.2 ±9.1	124.8 ±50.1	22.5 ±7.6	382.2 ±72.8	69.5 ±13.6	293.5 ±55.5	53.4 ±10.5	131.4 ±43.1	23.8 ±7.6
MT3	394.2 ±71.9	71.3 ±10.9	314.9 ±55.6	57.0 ±8.7	150.4 ±57.1	27.5 ±10.5	388.4 ±78.5	70.5 ±13.6	308.4 ±57.8	56.0 ±10.7	152.9 ±53.4	27.9 ±10.3
MT4	376.4 ± 63.0	68.1 ± 9.9	295.8 ± 50.5	53.5 ± 7.6	142.1 ±47.7	25.8 ± 8.0	372.8 ± 70.0	67.7 ±12.5	299.6 ±55.0	54.3 ±9.9	135.8 ±57.7	24.5 ±10.5
MT5	308.0 ±65.8	55.7 ±10.7	212.7 ±43.0	38.3 ±5.8	104.8 ±37.1	18.7 ±5.5	325.5 ±67.5	58.9 ±11.3	216.2 ± 44.4	38.9 ± 6.1	95.0 ±33.6	16.9 ± 4.6
MM	267.2 ±108.2	48.3 ±18.8	196.6 ±77.5	35.3 ±12.5	136.6 ±78.8	24.3 ±12.8	270.0 ±100.9	49.1 ±17.6	192.6 ±70.3	34.7 ±10.9	126.5 ±63.8	22.5 ±9.9
LM	353.6 ±66.3	63.8 ±9.5	222.3 ±44.6	40.2 ±7.2	82.2 ±20.8	14.8 ±2.3	377.7 ±79.8	68.1 ±10.9	241.2 ±46.8	43.8 ±7.9	84.0 ±63.8	15.0 ±2.8
T	368.7 ±79.0	66.2 ±9.6	343.8 ±72.7	61.8 ±9.6	415.1 ±90.3	57.3 ±13.5	373.1 ±90.2	66.9 ±11.1	347.3 ±81.6	62.3 ±10.5	319.1 ±88.4	57.3 ±13.3
MH	546.0 ±102.0	97.6 ±4.5	369.6 ±69.9	66.1 ±4.6	90.4 ±27.3	16.3 ±3.8	525.3 ±97.3	94.0 ±6.2	340.6 ±60.1	61.2 ±5.2	99.3 ±37.3	17.9 ±4.7
LH	534.2 ±102.9	95.5 ±5.6	349.7 ±68.1	62.6 ±5.2	102.0 ±38.5	18.2 ±4.8	527.4 ±97.8	94.4 ±6.1	354.5 ±67.4	63.5 ±5.3	91.8 ±25.6	16.4 ±3.5

Data are means ±SD.
MAX is the maximum plantar pressure, which is the major reference point averaged for a group irrespective of foot-sidedness.
GT, great toe; LT, lateral toes; MT, metatarsals 1-5; MM, medial midfoot; LM, lateral midfoot; T, tarsal; MH, medial hindfoot; LH, lateral hindfoot.
MxP, maximum pressure; AvP, average pressure; MnP, minimum pressure.

	Class 2 - WOMEN (n = 157)											
	MAX (g/cm^2) - 529.3 ±96.2											
	Left Foot						Right Foot					
	Plantar pressure data						Plantar pressure data					
Plantar zone	MxP (g/cm^2)	MxP (%MAX)	AvP (g/cm^2)	AvP (%MAX)	MnP (g/cm^2)	MinP (%MAX)	MxP (g/cm^2)	MxP (%MAX)	AvP (g/cm^2)	AvP (%MAX)	MnP (g/cm^2)	MnP (%MAX)
GT	196.7 ±82.1	39.0 ±18.7	138.5 ±53.5	27.2 ±11.9	77.6 ±34.9	14.8 ±6.2	196.1 ±72.7	38.4 ±15.9	136.5 ±46.6	26.5 ±9.6	80.0 ±29.7	15.3 ±5.2
LT	147.9 ±86.1	29.0 ±17.0	108.2 ±56.9	21.1 ±11.0	72.9 ±39.4	14.1 ±7.3	147.5 ±81.7	29.0 ±16.5	110.2 ±53.7	21.5 ±10.5	76.1 ±38.7	14.6 ±6.9
MT1	337.3 ±55.4	65.0 ±12.2	241.2 ±39.7	46.4 ±8.5	94.8 ±34.0	18.0 ±6.1	328.9 ±55.2	63.3 ±11.3	229.2 ±38.9	44.1 ±7.8	90.3 ±24.9	17.2 ±4.4
MT2	370.8 ±60.6	71.2 ±11.3	294.1 ±48.6	56.5 ±9.2	130.1 ±56.1	24.6 ±10.2	349.5 ±58.7	67.2 ±11.5	273.1 ±47.2	52.6 ±9.4	123.7 ±41.6	23.6 ±7.8
MT3	367.3 ±62.4	70.4 ±10.9	295.8 ±46.5	56.8 ±8.9	154.3 ±58.5	29.8 ±11.8	343.2 ±58.0	66.0 ±11.2	281.4 ±48.1	54.2 ±9.8	160.7 ±57.0	31.0 ±11.5
MT4	349.0 ±62.3	67.0 ±11.3	274.4 ±45.4	52.8 ±9.1	142.1 ±49.6	27.4 ±10.4	329.5 ±56.1	63.3 ±10.7	263.0 ±45.2	50.5 ±8.6	138.4 ±54.9	26.3 ±10.2
MT5	274.8 ±66.5	52.8 ±13.0	192.4 ±40.7	36.8 ±7.1	100.5 ±31.1	19.1 ±5.6	270.9 ±69.9	52.0 ±13.2	182.9 ±45.3	34.8 ±7.4	91.0 ±31.5	17.1 ±4.8
MM	238.2 ±128.8	44.8 ±22.6	176.3 ±90.4	33.1 ±15.5	120.7 ±74.7	22.6 ±13.4	262.0 ±103.6	49.6 ±18.0	179.0 ±63.2	33.9 ±10.7	107.4 ±48.4	20.5 ±9.1
LM	336.7 ±82.7	64.0 ±11.6	210.9 ±51.9	40.2 ±7.8	78.2 ±22.1	14.7 ±2.4	335.9 ±65.7	64.4 ±11.1	211.2 ±42.2	40.5 ±7.6	80.0 ±20.1	15.2 ±3.2
T	353.5 ±79.9	66.9 ±9.7	334.6 ±73.6	63.4 ±8.7	313.2 ±78.5	59.3 ±10.8	346.9 ±79.3	65.9 ±10.8	328.5 ±72.6	62.4 ±9.9	308.9 ±75.7	58.6 ±11.4
MH	512.1 ±98.7	96.7 ±5.6	340.0 ±66.8	64.2 ±4.5	89.3 ±28.0	17.0 ±4.7	493.1 ±91.4	93.4 ±6.7	314.7 ±56.0	59.7 ±4.8	91.9 ±29.2	17.4 ±4.7
LH	499.1 ±96.5	94.3 ±6.5	323.3 ±59.2	61.3 ±4.8	97.6 ±31.8	18.5 ±5.4	495.5 ±91.5	93.8 ±6.5	332.2 ±61.1	63.0 ±5.4	89.5 ±30.7	16.9 ±4.5

Data are means ±SD.
MAX is the maximum plantar pressure, which is the major reference point averaged for a group irrespective of foot-sidedness.
GT, great toe; LT, lateral toes; MT, metatarsals 1-5; MM, medial midfoot; LM, lateral midfoot; T, tarsal; MH, medial hindfoot; LH, Lateral hindfoot.
MxP, maximum pressure; AvP, average pressure; MnP, minimum pressure.
See further details in the manuscript's body text.
